# Enhanced Chimeric Antigen Receptor T Cell Therapy through Co-Application of Synergistic Combination Partners

**DOI:** 10.3390/biomedicines10020307

**Published:** 2022-01-28

**Authors:** Sophia Stock, Anna-Kristina Kluever, Stefan Endres, Sebastian Kobold

**Affiliations:** 1 Division of Clinical Pharmacology, Department of Medicine IV, University Hospital, Ludwig Maximilian University (LMU) of Munich, 80337 Munich, Germany; A.Kluever@campus.lmu.de (A.-K.K.); Stefan.Endres@med.uni-muenchen.de (S.E.); 2 Department of Medicine III, University Hospital, Ludwig Maximilian University (LMU) of Munich, 81337 Munich, Germany; 3 German Center for Translational Cancer Research (DKTK), Partner Site Munich, 80336 Munich, Germany; 4 Einheit für Klinische Pharmakologie (EKLiP), Helmholtz Zentrum München, German Research Center for Environmental Health (HMGU), 85764 Neuherberg, Germany

**Keywords:** chimeric antigen receptor, adoptive T cell therapy, combination therapies

## Abstract

Chimeric antigen receptor (CAR) T cell therapy has achieved remarkable response rates and revolutionized the treatment of patients suffering from defined hematological malignancies. However, many patients still do not respond to this therapy or relapse after an initial remission, underscoring the need for improved efficacy. Insufficient in vivo activity, persistence, trafficking, and tumor infiltration of CAR T cells, as well as antigen escape and treatment-associated adverse events, limit the therapeutic success. Multiple strategies and approaches have been investigated to further improve CAR T cell therapy. Besides genetic modification of the CAR itself, the combination with other treatment modalities has the potential to improve this approach. In particular, combining CAR T cells with clinically approved compounds such as monoclonal antibodies and small molecule inhibitors might be a promising strategy. Combination partners could already be applied during the production process to influence the cellular composition and immunophenotype of the final CAR T cell product. Alternatively, simultaneous administration of clinically approved compounds with CAR T cells would be another feasible avenue. In this review, we will discuss current strategies to combine CAR T cells with compounds to overcome recent limitations and further enhance this promising cancer therapy, potentially broadening its application beyond hematology.

## 1. Introduction

Current therapeutic approaches in cancer therapy are developing quickly, and immunotherapeutic strategies are becoming more and more important. In particular, the adoptive transfer of genetically engineered T cells, namely chimeric antigen receptor (CAR)-modified T cells, has achieved remarkable response rates and has revolutionized the treatment of certain hematological malignancies. Anti-CD19 and anti-BCMA CAR T cells showed the most promising results leading to clinical approval. For anti-CD19 CAR T cells, the European Medicines Agency (EMA) and the U.S. Food and Drug Administration (FDA) approved the product Yescarta^®^ (Axicabtagene ciloleucel) for patients with relapsed/refractory (r/r) diffuse large B cell lymphoma (DLBCL) and primary mediastinal B cell lymphoma (PMBCL) based on the results of the ZUMA-1 trial [1], as well as the product Kymriah^®^ (Tisagenlecleucel) for the treatment of patients with r/r B cell acute lymphoblastic leukemia (ALL) based on the ELIANA trial [2] and for patients with DLBCL based on the JULIET trial [3]. The product Tecartus^®^ (Brexucabtagene autoleucel) was approved for the therapy of r/r mantle cell lymphoma (MCL) based on the results of the ZUMA-2 trial [4]. Due to the TRANSCEND trial, the CD19-directed CAR T cell product Breyanzi^®^ (Lisocabtagene maraleucel) was approved for the treatment of r/r large B cell lymphoma (LBCL) [5]. The most recently approved CAR T cell product was Abecma^®^ (Idecabtagene vicleucel) for the treatment of r/r multiple myeloma (MM) based on the results of the KarMMa trial [6,7]. Other tumor target antigens for CAR T cell therapy are currently under development but have not yet been granted approval for clinical use.

Even though CAR T cell therapy has reached the clinic on a commercial basis and has shown very promising clinical results, therapeutic failure and relapse can still be observed. Gaining a better understanding of the reasons underlying treatment failure will enable the development of strategies to overcome the current limitations of this therapeutic approach. Therapeutic success is limited—among others—by insufficient in vivo persistence, activation, migration, and tumor infiltration of CAR T cells, as well as by therapy-associated toxicities such as cytokine release syndrome (CRS), immune effector cell-associated neurotoxicity syndrome (ICANS), on-target off-tumor toxicities, and antigen loss [8,9,10,11,12]. However, it is important to distinguish intrinsically poor T cell functionality from induced CAR T cell dysfunction in vivo due to the tumor microenvironment. To further improve CAR T cell therapy, multiple strategies and approaches have been investigated, including altering the composition of the CAR itself and target antigens. Besides this genetic modification, the combination of CAR T cells with other treatment modalities such as chemotherapy, radiotherapy, and non-cellular immunotherapy might have the potential to improve this promising therapeutic approach. In particular, combination of CAR T cells with clinically approved compounds such as monoclonal antibodies or small molecule inhibitors might be a promising strategy to positively influence the activity and properties of CAR T cells. This can be performed in vitro during CAR T cell production to generate a more potent CAR T cell product and in vivo as a preconditioning regime or as a synergistic combination therapy. Since the proliferative capacity of CAR T cells, and therefore, their in vivo efficacy, is influenced by the cellular composition and phenotype of the final cell product [13,14,15], including a small molecule inhibitor during CAR T cell production to enhance the final CAR T cell product could be advantageous. Alternatively, applying special preconditioning regimes or simultaneously administering clinically approved compounds with CAR T cells could also be feasible approaches to improve the anti-tumor efficacy of CAR T cells.

In this review, we will discuss preclinical and clinical strategies currently used to combine CAR T cell therapy in vitro or in vivo with antibodies, small molecule inhibitors, and other preclinically or clinically approved compounds with the aim of overcoming the current limitations of this very promising therapeutic approach.

## 2. CAR T Cell Therapy

### 2.1. CAR T Cell Production Process

**Figure 1:**
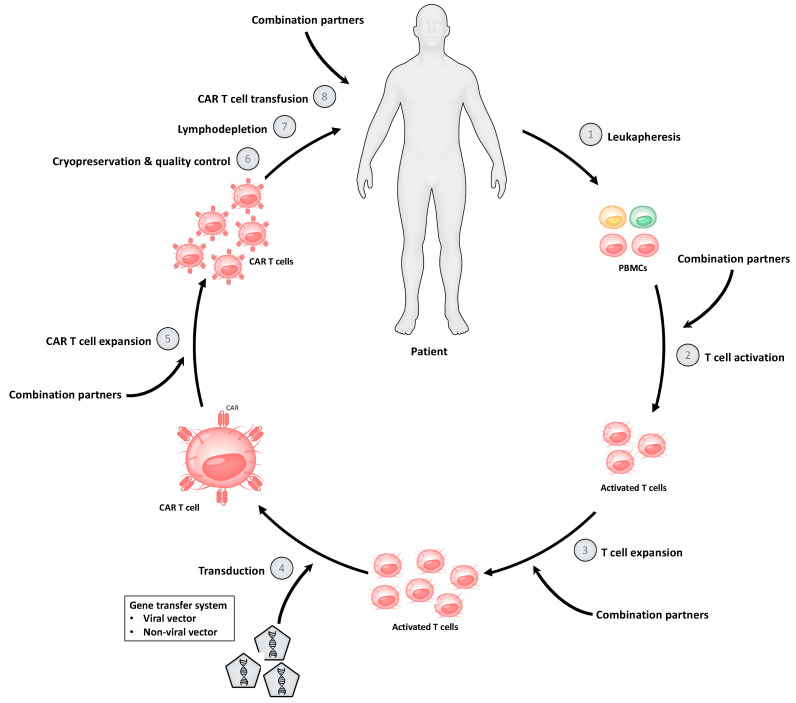
Principle of the chimeric antigen receptor (CAR) T cell manufacturing process. The process starts with leukapheresis for isolation of peripheral blood mononuclear cells (PBMCs). The next step includes activation and expansion of T cells until transduction with the CAR vector is performed followed by expansion of the CAR T cells. After end-of-process formulation, quality checks, and cryopreservation, the final CAR T cell product can be administered into the patient after a lymphodepleting chemotherapy. Combination partners can be used for ex vivo treatment of CAR T cells during the production process or for simultaneous administration with CAR T cells into the patient.

As an increasing variety of CAR T cell products is being developed, the CAR T cell manufacturing process is also becoming more diverse. However, most of the important steps of the CAR T cell production are typically shared between cellular products (Figure 1). The first step of the manufacturing process includes the isolation and enrichment of CD3+ T cells. Peripheral blood mononuclear cells (PBMCs) are commonly obtained from peripheral blood of a patient, and T cells are subsequently isolated by density gradient centrifugation or automated cell-washers [16,17]. In patients with a high tumor burden in the peripheral blood, additional selection or depletion of specific T cell subtypes might be necessary to expand and administer a CAR T cell product with a defined cellular composition [17]. An indispensable step of the manufacturing process is the activation of T cells, which aims to promote sufficient T cell expansion without inducing terminal differentiation or activation-induced cell death (AICD). The most common T cell activation strategies include anti-CD3 monoclonal antibodies (OKT-3) with or without anti-CD28 monoclonal antibodies, as well as anti-CD3 and anti-CD28 antibody-coated magnetic beads [16]. Beads can mediate an ongoing selection and activation of CD3+ T cells until the CAR T cell production is ready for application. After the initial activation and expansion of T cells, a non-viral or viral gene transfer of the vector with the corresponding genetic information is performed. Gammaretroviral or lentiviral vectors are most commonly used. However, plasmid-based transposon/transposase systems and genome engineering tools such as CRISPR/-Cas9-based gene editing have become more and more popular [17]. After successful transduction of the CAR vector, CAR T cells are expanded for several days to increase the total amount of cells until cryopreservation is performed. Ex vivo expansion of CAR T cells is usually performed in the presence of cytokines, especially interleukin (IL)-2, IL-7 and IL-15 [16]. The addition of stimulating cytokines during the manufacturing process represents an indispensable and important step with a major impact on the quality and quantity of the final CAR T cell product [16].

After sufficient CAR T cell expansion at the end of the production process, the final product is cryopreserved. This enables the long-distance transportation of the CAR T cell product from the manufacturing site to clinical centers. Currently, transportation is a relevant burden, as only a few manufacturing sites exist, and these therapies are applied worldwide. Moreover, before administration of the CAR T cell product, final quality control tests are mandatory. Patients usually receive a lymphodepleting chemotherapy before CAR T cell therapy is applied.

### 2.2. CAR T Cell Construct

**Figure 2:**
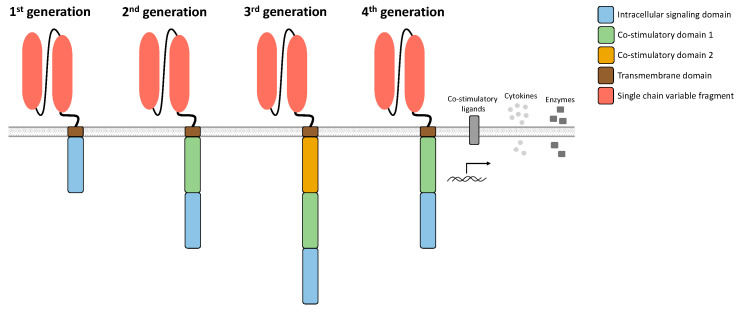
Composition of the chimeric antigen receptor. The receptor consists of single chain variable fragment for extracellular antigen recognition, a non-signaling spacer, a transmembrane domain, optional co-stimulatory domains, and a CD3-zeta chain as intracellular signaling domain.

CAR T cells are redirected to bind and eradicate target antigen-expressing malignant cells through the expression of engineered synthetic receptors. A prerequisite for efficient CAR T cell therapy is the optimal composition of the CAR construct. Its typical composition (Figure 2) consists of a single chain variable fragment (scFv) of an antibody, a non-signaling extracellular spacer, a transmembrane (TM) domain, and a CD3-zeta (CD3ζ) chain for intracellular signaling [18]. The scFv serves as an extracellular binding domain for human leukocyte antigen (HLA)-independent recognition of the target antigen [18]. The spacer located between the TM domain and the scFv usually consists of an immunoglobin G (IgG)-based hinge domain [12] and also has an important effect on CAR T cell functionality [12,19,20]. Antigen-independent activation of the CAR, so-called tonic CAR signaling, is mediated by certain spacers; however, linker length and the scFv part also play an important role [12]. CAR design has changed and developed in recent decades since its first description (Figure 2).

So-called first-generation CAR T cells did not have a co-stimulatory domain, so that T cell activation was mediated exclusively by the intracellular CD3ζ chain. First-generation CAR T cells mediated cytotoxicity, but with poor expansion, cytokine production, and survival [18,21,22]. Second-generation CAR T cells integrated a co-stimulatory domain such as CD28 or CD137 (4-1BB) to improve T cell persistence and expansion and to prevent anergy and AICD, thus more closely mimicking physiological T cell activation [16]. So far, second-generation CAR T cells represent the only FDA-approved class of CAR T cell products. A further advance in CAR design was the integration of two co-stimulatory domains [16]. For these third-generation CAR T cells, efficacy and safety could be shown in patients with B cell malignancies treated with CD19-specific CAR T cells [23]. Later, fourth-generation CAR T cells were developed, which can co-express additional molecules besides the CAR construct in suitable vectors [24]. These include T cells redirected for universal cytokine-mediated killing (TRUCKs), which are CAR-redirected vehicles with the ability to produce and release an inducible product such as a specific cytokine [24].

Currently, an enormous effort is being put into further optimizing the CAR composition. Most preclinical and clinical data generated with CAR T cells rely on second-generation CAR T cells followed by third-generation CAR T cells. New insights into CAR T cell therapy will be gained when future CAR T cell generations reach large-scale clinical testing.

### 2.3. Current Limitations of CAR T Cell Therapy

**Figure 3:**
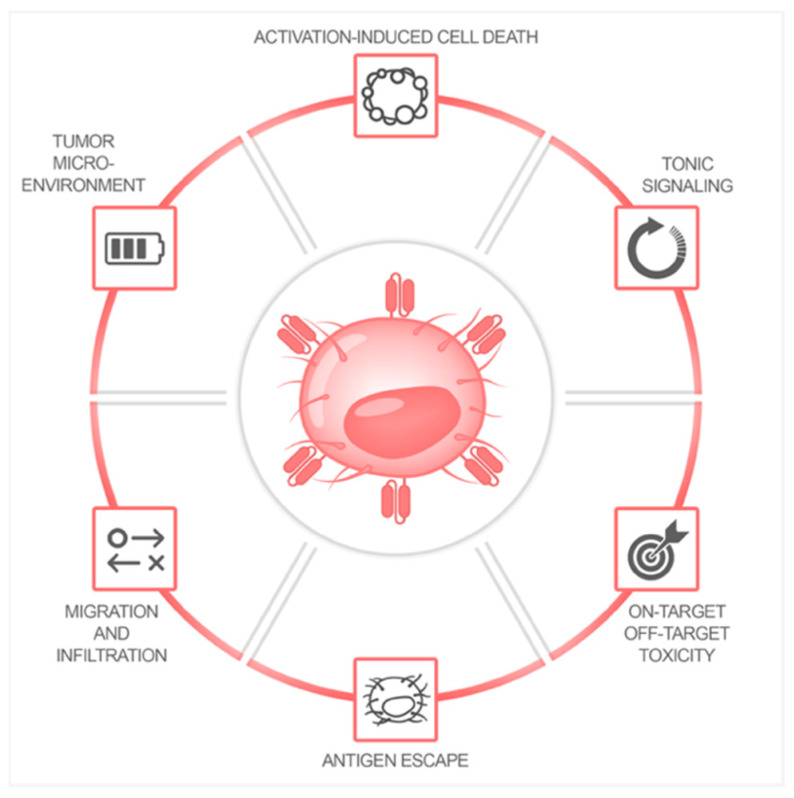
Current limitations of CAR T cell therapy.

CAR T cell therapy is well established in certain hematological diseases. However, even in these settings, therapeutic failure and relapse can occur. In solid tumors, the efficacy of CAR T cell therapy remains to be demonstrated [25]. Therapeutic failure can depend on the composition of the CAR construct including the antigen binding domain, spacer, transmembrane domain, and intracellular co-stimulatory domains [12,19,20,26,27]. Moreover, AICD and antigen-independent tonic signaling can limit the response rate of CAR T cell therapy [12]. Tonic CAR signaling leads to early exhaustion of CAR T cells and therefore limits CAR T cell functionality [28]. Additionally, toxicities associated with CAR T cells such as CRS and ICANS, as well as on-target off-tumor toxicities, restrict the clinical feasibility and application of this cellular immunotherapy [29]. Optimization of the target antigen selection is particularly important in solid tumor models, as many tumor antigens are also expressed on healthy tissues, leading to severe on-target off-tumor toxicity. Even if CAR T cell therapy was initially successful, antigen-negative relapse can still be observed in a proportion of patients over time [11,12,29]. Improving the choice of target antigens can thus not only enhance anti-tumor efficacy but might also prevent the occurrence of antigen-negative relapse. In addition, in the setting of solid tumors, insufficient migration and infiltration into the tumor tissue can also hinder the therapeutic efficacy of CAR T cells compared to their application in hematological diseases [11,25]. Tumor stroma serves as a physical tumor barrier, and the immunosuppressive microenvironment hinders CAR T cells from successfully infiltrating into the tumor site. Regional delivery [30,31] and co-expression of fibroblast activation protein (FAP) [32], heparanase enzyme [33], or specific chemokine receptors [34,35,36,37] on CAR T cells represent potential strategies to improve migration and infiltration. Additional strategies have been developed to shield CAR T cells from inhibitory signals of the tumor microenvironment such as the co-expression of a dominant-negative receptor (DNR) for transforming growth factor beta (TGF-β) [37,38,39,40]. Besides these genetic modifications, the combination of other treatment modalities with adoptive T cell therapy may increase the success of this promising therapy [41,42,43,44]. More modern therapeutic approaches such as targeted therapies with monoclonal antibodies or small molecule inhibitors have become increasingly interesting as potential combination partners with cellular immunotherapy [45]. Limitations of CAR T cell therapy (Figure 3) and possible targets for optimization have been extensively reviewed elsewhere [11,12,25,29].

## 3. Combination Partners for Ex Vivo CAR T Cell Treatment 

During the ex vivo manufacturing process, CAR T cells are expanded under supplementation of specific γ-chain cytokines such as IL-2, IL-7 and/or IL-15, influencing the phenotype and cellular composition of the cell product at the end of the production process [16]. Less well known is the fact that CAR T cell production can also be performed in the presence of these cytokines and small molecule inhibitors or other drugs to further improve the quality and quantity of the final CAR T cell product (Table 1).

**Figure 4:**
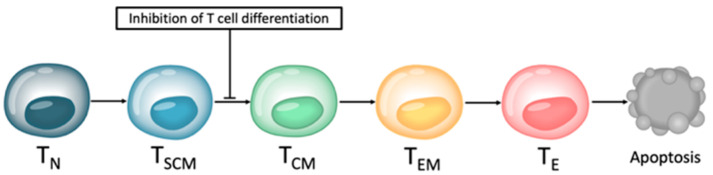
Interruption of T cell differentiation. Small molecules inhibiting key metabolic and developmental pathways might cause an interruption of the T cell differentiation process from naïve-like T (T_N_) cells and stem cell memory-like T (T_SCM_) cells towards T central memory-like (T_CM_) cells, T effector memory-like (T_EM_) cells, and terminally differentiated T effector-like (T_E_) cells.

Small molecule inhibitors are designed to target a specific part of a molecule. They can penetrate the cell membrane and therefore inhibit intracellular molecules due to their very small size. The majority of these small molecule inhibitors target signaling pathways by blocking tyrosine kinases or serine-threonine kinases that are involved in tumor growth, angiogenesis, and metastasis. Not as well known is the fact that they might also influence T cells and other non-malignant cells independently of their effect on tumor cells. Ex vivo generation and expansion of genetically modified T cells in the presence of specific signaling pathway inhibitors could lead to an interruption of the T cell differentiation process (Figure 4). Thus, the immunophenotype and cellular composition of the T cell product at the end of the production process could shift towards a less differentiated phenotype with more naïve-like T (T_N_) and stem cell memory-like T (T_SCM_) cells [62].

### 3.1. Protein Kinase Inhibitors

Protein kinase inhibitors including inhibitors of the PI3K-Akt-mTOR pathway and of Bruton’s tyrosine kinase (BTK) have shown promising effects on CAR T cells when used for ex vivo treatment of CAR T cells during the production process (Table 1).

#### 3.1.1. Inhibitors of the PI3K-Akt-mTOR Signaling Pathway

The activation, function, differentiation, survival, expansion, and migration of T cells are—among others—influenced by the PI3K-Akt-mTOR signaling pathway [63]. mTOR signaling plays an essential role for both T cells and tumor cells. In a preclinical setting, the mTOR inhibitor rapamycin could promote an increase in T memory cells with a higher expression of the anti-apoptotic molecule Bcl-2 and the lymph node homing marker L-Selectin (CD62L) [46]. IL-15-mediated suppression of mTORC1 activity led to a less differentiated CAR T cell phenotype during the generation process [64]. Additionally, CAR T cells expanded in vitro in the presence of IL-2 and the mTORC1 inhibitor rapamycin also maintained a less differentiated T cell phenotype, an effect most likely mediated by decreased mTORC1 activity [64]. Furthermore, mTORC1 signaling in CAR T cells led to diminished infiltration of CAR T cells into the bone marrow [47]. Anti-EpCAM CAR T cells treated ex vivo during T cell expansion with rapamycin showed an enhanced bone marrow infiltration and leukemia elimination in an AML xenograft mouse model [47].

From another perspective, inhibition of the serine-threonine kinase Akt can be used to influence T cell products. Ex vivo expansion of tumor-infiltrating lymphocytes (TILs) in the presence of the Akt inhibitor VIII mediated potent tumor-specific T cells with memory cell characteristics [48]. This led to an increased in vivo persistence and anti-tumor effector function in an immunodeficient mouse model [48]. Along these lines, ex vivo Akt inhibition can lead to CAR T cells or T cell receptor (TCR)-modified T cells with a central memory-like T (T_CM_) cell phenotype and high CD62L expression [49,50]. Inhibition of Akt signaling led to MAPK activation and promoted a transcriptional regulator of T cell memory called FOXO1 [49]. Anti-CD19 CAR T cells treated with an Akt inhibitor ex vivo showed an enhanced in vivo anti-tumor efficacy compared to conventionally expanded T cells [49,50]. Additionally, disialoganglioside (GD2)-specific CAR T cells co-expressing transgenic constitutively active Akt (caAkt) were resistant to tumor-associated inhibitory mechanisms after co-culture with GD2-positive tumor cells [65]. Anti-GD2 CAR T cells showed increased proliferation, persistence, and cytokine production [65]. These results underline the potential of Akt inhibitors for combination with T cell-based therapies.

Inhibition of phosphatidylinositol-3-kinase (PI3K) might also influence the final CAR T cell product. Pre-treatment of CAR T cells with a PI3K inhibitor during the production process promoted less differentiated T cells with a high CCR7 and CD62L expression along with enhanced effector functions in anti-CD19 [51], anti-mesothelin [52,53], and anti-CD33 [54] CAR T cells. In the presence of idelalisib (CAL-101), a PI3Kδ inhibitor used for the treatment of chronic lymphocytic leukemia (CLL) and follicular lymphoma (FL), CLL patient-derived CAR T cells acquired a more balanced CD4+ T cell to CD8+ T cell ratio [51]. A less exhausted phenotype of CAR T cells was also promoted by in vitro treatment of CAR T cells with idelalisib [51,52]. Another very promising approach for T cell immunotherapies is targeting PI3Kγ [66]. Anti-mesothelin CAR T cells cultivated with the PI3Kγ inhibitor eganelisib (IPI-549), PI3Kδ inhibitor idelalisib (CAL-101), or dual PI3Kδ/CK1ε inhibitor umbralisib (TGR-1202) had superior in vivo potency and a less differentiated phenotype [53]. However, treatment of anti-mesothelin CAR T cells with the dual PI3Kγ and PI3Kδ inhibitor duvelisib (IPI-145) led to reduced effector functions [53]. Interestingly, CAR T cells treated with a PI3Kδ inhibitor had superior in vitro cytotoxicity compared to CAR T cells treated with a PI3Kγ inhibitor or conventionally expanded CAR T cells [53]. In addition, CAR T cells expanded ex vivo with duvelisib had acquired a less differentiated phenotype with a higher mitochondrial mass, mediating improved in vivo engraftment, expansion, tumor eradication, and mouse survival in an immunodeficient NOD/Shi-scid/IL-2Rγ^null^ (NOG) mouse model [55]. Interestingly, duvelisib could decrease the secretion of IL-6 and therefore the occurrence of CRS in an anti-CD19 CAR T cell model [67]. A clinical phase I trial investigated the influence of ex vivo expansion of the anti-BCMA CAR T cell product bb21217 (based on ide-cel) in the presence of the PI3K inhibitor bb007. The product had fewer CD57+ senescent cells, an increased CD127 expression, more CD27+ CCR7+ T memory cells, and improved in vivo proliferation [56]. Another combinatorial approach during the in vitro expansion of DLBCL patient-derived T cells is the application of antagonists of vasoactive intestinal peptide (VIP) receptor and of PI3Kδ [57]. An interruption of T cell differentiation with reduced PD-1 expression and enhanced in vivo persistence were mediated by these antagonists [57]. Ex vivo treatment of anti-CD5 CAR T cells with these antagonists promoted an enhanced proliferative capacity, transduction efficiency and anti-tumor effector function against CD5+ lymphoma cells [57]. Another interesting target could be the B cell adaptor for PI3K (BCAP), which also affects the differentiation of CD8+ T cells [68].

These mostly preclinical data demonstrate that the ex vivo treatment of CAR T cells in the presence of small molecule inhibitors interacting with PI3K-Akt-mTOR signaling pathway could be beneficial (Table 1). In any case, clinical studies are required to better understand this approach and the consequences for CAR T cell patients.

#### 3.1.2. BTK Inhibitors

The irreversible BTK inhibitor ibrutinib is clinically approved for the treatment of CLL and MCL. Its positive influence on T cells is less well known. By inhibiting interleukin-2-inducible T cell kinase (ITK), it can influence T cell differentiation [69]. Ex vivo treatment of anti-CD19 CAR T cells with ibrutinib during the production process led to improved T cell viability and proliferative capacity with higher T cell numbers and mediated a less differentiated T cell phenotype [58]. Additionally, ibrutinib mediated reduced exhaustion marker expression on CAR T cells, underscoring an additional advantage of this preclinical combinatorial approach [58]. Clinical evaluation will further show if this strategy can improve CAR T cell therapy.

### 3.2. Epigenetic Modulators

Epigenetic modulators for ex vivo expansion of genetically modified T cells represent another promising strategy. JQ1 is an inhibitor of the epigenetic modulator BRD4, which is a member of the bromodomain and extra-terminal motif (BET) subfamily of human bromodomain proteins. BRD4 regulates the expression of the transcription factor BATF in cytotoxic T cells, which is involved in differentiation into a T_EM_ cell phenotype [59]. Treatment of T cells with JQ1, promoted the expansion of less differentiated T_SCM_ and T_CM_ cells and enhanced persistence and effector function in murine TCR and CAR gene therapy models [59]. Downregulation of c-Myc-dependent target genes by this BET bromodomain inhibitor could be a reason for these phenotypic and functional changes [70]. Anti-CD33 CAR T cells treated for 4 days with the BET inhibitors JQ-1 or iBET 5 days after activation exhibit an increase in less differentiated T cells [60]. This approach needs to be confirmed in clinical trials to further understand its potential.

### 3.3. Immunomodulatory Drugs

Primary application of immunomodulatory drugs such as thalidomide and its analogues lenalidomide, pomalidomide, and iberdomide is the treatment of autoimmune diseases and cancer, notably of MM. T cells play a known important role in the overall anti-myeloma effects of these immunomodulatory drugs [71]. Lenalidomide is widely used in the treatment of MM [72]. It can also be used to treat CAR T cells during the ex vivo production process. Lenalidomide-treated anti-CS1 CAR T cells acquired a memory phenotype as well as an enhanced killing capacity, cytokine secretion of T helper (T_H_) 1 cells and immune synapse formation [61]. In vitro treatment of anti-CS1 CAR T cells with lenalidomide also improved anti-tumor efficacy and T cell persistence in an in vivo model [61]. Clinical data are required to further assess the use of this new CAR T cell production approach.

## 4. Synergistic Combination Therapy with CAR T Cells

Several strategies are recently underway to further optimize CAR T cell efficacy and to decrease treatment-associated toxicity. The combination of cellular immunotherapy with targeted therapies has recently taken on greater significance for adoptive T cell therapies. While monoclonal antibodies target specific cell surface antigens, small molecule inhibitors enter the cell and interfere with or inhibit the enzymatic activity of specific intracellular proteins, which are involved in important signaling pathways. Several interesting combination partners for synergistic co-application with CAR T cells have been investigated in pre-clinical and clinical settings in recent years (Table 2). Most of the data, however, have been generated in pre-clinical trials. Profound clinical data are still missing for the majority of these combinatorial approaches.

### 4.1. Immune Checkpoint Modulators

Application of immune checkpoint modulators, especially of immune checkpoint inhibitors, is one of the most promising therapeutic approaches in modern cancer therapy. Exhausted T cells can be reactivated through immune checkpoint blockade with long-lasting response rates; however, for very immunogenic cancer entities such as melanoma, responses rates are less than 40% [121]. A prerequisite for the success of checkpoint blockade therapy is a pre-existing anti-tumor immune response prior to therapy, which can be reactivated by checkpoint inhibitors [122]. Therefore, the combination of checkpoint blockade therapy with a different type of immunotherapy with the ability to mediate an immunogenic tumor microenvironment or an antigen-specific T cell product could possibly overcome the current limitations of checkpoint blockade therapy [123]. In particular, the combination with CAR T cells is very interesting in hematological and solid tumor models. Administered CAR T cells might infiltrate an immunogenically silent tumor, and the consequent CAR T cell exhaustion and inhibition might be reversed by immune checkpoint blockade. Effector functions of CAR T cells are reduced by an overexpression of PD-L1 and PD-L2 on malignant cells, underscoring the potential of a combination with immune checkpoint inhibitors [124]. Immune checkpoint modulators used for combination with CAR T cells are mostly monoclonal antibodies targeting PD-1, PD-L1, CTLA-4, and 4-1BB (Table 2).

#### 4.1.1. Antibodies Targeting PD-1, PD-L1, and CTLA-4

In a preclinical model of mice bearing two different HER2-positive tumor models, tumor control was enhanced when anti-HER2 CAR T cells were administered together with an anti-PD-1 antibody without causing autoimmune pathology in healthy HER2-expresssing tissue [73]. Combination of anti-GD2 CAR T cells with pembrolizumab also enhanced CAR T cell function and survival after repeated antigen stimulation, mediating improved cytotoxicity against PD-L1 expressing tumor cells [74]. PD-1 checkpoint blockade has the potential to rescue CAR T cell effector function after exhaustion, but only in the presence of the antibody, making repeated antibody administration essential [124]. In a clinical phase I trial, a patient with DLBCL of primary mediastinal origin was treated with anti-CD19 CAR T cells, and starting on day 26 after CAR T cell infusion, pembrolizumab was administered, leading to a decrease in PD-1-expressing CAR T cells, improved T cell expansion, and tumor regression [75]. Some patients with r/r non-Hodgkin lymphoma (NHL) treated with pembrolizumab after failure of anti-CD19 CAR T cells demonstrated a subsequent CAR T cell re-expansion [76]. It could also be shown that PD-1 inhibition has the potential to enhance anti-CD19 CAR T cell functionality in ALL patients [77]. A phase I study with neuroblastoma patients, however, revealed no clear benefit from the addition of pembrolizumab to anti-GD2 CAR T cell therapy [78]. Another clinical trial treated malignant pleural mesothelioma patients with anti-mesothelin CAR T cells together with pembrolizumab and demonstrated that the approach is safe and feasible. Moreover, they showed evidence of anti-tumor efficacy in patients with malignant pleural diseases [79]. A single-center study demonstrated the efficacy and safety of a combined treatment with anti-CD19 CAR T cells and the anti-PD-1 antibody nivolumab in 11 patients with r/r B cell NHL [80]. Furthermore, a successful co-treatment with anti-CD19 CAR T cells and nivolumab was reported for a patient with refractory FL [81]. In the ZUMA-6 clinical trial, the anti-CD19 CAR T cell product axi-cel combined with the anti-PD-L1 monoclonal antibody atezolizumab for the treatment of r/r DLBCL showed overall feasibility; however, cases of severe CRS and ICANS were reported [82]. Retrospective evaluation of CAR T cell numbers in the ZUMA-1 trial showed that patients who also received atezolizumab in the ZUMA-6 trial had an improved CAR T cell proliferative capacity [82]. Another trial aimed to treat patients with r/r aggressive B cell NHL with anti-CD19 CAR T cells of a defined composition (JCAR014) in addition to the PD-L1 antibody durvalumab prior to or after CAR T cell administration [83]. Complete responses were observed both at initial restaging after administration of the CAR T cells and in patients receiving and continuing with durvalumab therapy after not initially having achieved a complete remission [83]. Another trial treating intermediate or low-grade NHL, ALL, or CLL with anti-CD19 CAR T cells and the CTLA-4-directed antibody ipilimumab 2 weeks after T cell injection (NCT00586391) is still in progress.

Despite these promising results, the combination of CAR T cells with immune checkpoint inhibitors still requires further investigation. In particular, more long-term follow-up data and larger patient cohorts are required to fully understand the clinical potential of this combination strategy for future CAR T cell therapy.

#### 4.1.2. Antibodies Targeting 4-1BB

Another group of important immune checkpoint modulators are monoclonal antibodies against 4-1BB (CD137). 4-1BB (CD137) plays an important role in T cell proliferation, survival, and cytokine production. Activation of the 4-1BB (CD137) pathway by monoclonal antibodies can increase T cell responses [125]. In a human-HER2 self-antigen mouse model, the combination of anti-HER2 CAR T cells with an anti-4-1BB antibody reduced the amount of host immunosuppressive cells such as myeloid-derived suppressor cells and T_reg_ cells, leading to superior therapeutic efficacy in two solid tumor models [84]. The monoclonal antibody utomilumab (PF-05082566) can bind human 4-1BB and has already been tested in clinical trials for cancer therapy [126,127]. The ZUMA-11 phase 1/2 multicenter study testing the combination of the CD19-specific CAR T cell product axi-cel with utomilumab in r/r LBCL patients is currently in progress [85]. Detailed reports of these results will further establish the benefits of this combinatorial approach.

Despite these promising results, the combination of CAR T cells with immune checkpoint blockade still requires further investigation. In particular, more long-term follow-up data and larger patient cohorts are required to fully understand the clinical potential of this combination strategy for future CAR T cell therapy.

### 4.2. Immunomodulatory Drugs

Immunomodulatory drugs such as thalidomide, lenalidomide, pomalidomide, and iberdomide are well established in the treatment of cancer and autoimmune diseases. Immunomodulators are efficient in treating MM by directly targeting myeloma cells but also by mediating an anti-myeloma immune response [71].

The oral immunomodulatory drug lenalidomide is well established in the therapy of MM [72]. Lenalidomide influences T cell immunophenotype and functionality [128,129]. Analysis of lenalidomide-treated MM patient samples revealed a maturated T cell phenotype with decreased CD57 expression [128]. In vitro incubation of myeloma-specific T cells with lenalidomide mediated enhanced efficacy [128]. Therefore, lenalidomide is an interesting combination partner for CAR T cell therapy (Table 2). Co-application of lenalidomide enhanced the in vitro cytokine production and cytotoxicity of anti-BCMA CAR T cells compared to conventional CAR T cells [86]. In a mouse model, the survival and persistence of anti-BCMA CAR T cells in peripheral blood were improved in the presence of lenalidomide [86]. A clinical case report of a patient treated with lenalidomide one day prior to the administration of anti-BCMA CAR T cells demonstrated the combination to be feasible and effective [130]. Another antigen suitable for CAR T cell therapy in MM is CS1. CS1 is a cell surface glycoprotein of the signaling lymphocyte activation molecule (SLAM) receptor family, which has a high and selective expression on healthy plasma cells as well as malignant MM cells and is not expressed on other healthy tissues [131]. In a NOD/Scid IL2RγC^null^ mouse model, mice were treated with anti-CS1 CAR T cells and lenalidomide intraperitoneally daily for 30 days, leading to stronger tumor clearance and T cell persistence [61]. The positive effect of the combination therapy could also be seen with anti-CD20 and anti-CD19 CAR T cells in in vitro and in vivo experiments [87]. In the ongoing phase 2, open-label, multicenter ZUMA-14 trial (NCT04002401), patients with r/r LBCL are being treated with the anti-CD19 CAR T cell product axi-cel combined with either rituximab (Cohort 1) or lenalidomide (Cohort 2) [88]. In solid tumor models, a combination therapy of lenalidomide with CD133-specific CAR T cells led to enhanced in vitro cytotoxicity against the CD133 overexpressing human glioma cell line U251 and to increased proliferation of CAR T cells [89]. The killing capacity of anti-HER2 CAR T cells against the human breast cancer cell line MDA-MB-453 was also enhanced with lenalidomide [89]. The anti-tumor effect of CAR T cells against epidermal growth factor receptor variant III (EGFRvIII) expressed on glioblastoma multiforme was also enhanced by lenalidomide in an intracranial xenograft model [90]. Interestingly, lenalidomide mediated improved immunological synapses between tumor cells and T cells [90]. Furthermore, a combination of CAR T cells recognizing the WT1_235–243_ peptide with lenalidomide also showed enhanced tumor infiltration in a mouse model [91]. Superior efficacy was analyzed with proteomics studies identifying a lenalidomide-mediated effect on T cell activation, mitochondrial activity, and immune synapse formation [91].

These pre-clinical results indicate that lenalidomide is a very promising combination partner for various CAR T cells, not only in MM models but also in other hematological and solid tumor. Clinical results are necessary to further analyze the effects of this combinatorial approach.

### 4.3. Protein Kinase Inhibitors

The majority of clinically approved small molecule drugs inhibit important protein kinases such as tyrosine kinases or serine-threonine kinases and therefore target essential signal transduction mechanisms involved in tumor survival and growth [45]. These protein kinase inhibitors might influence physiological processes in non-malignant cells through off-target effects [132]. A positive influence on T cells mediated by protein kinase inhibitors seems promising for T cell-based immunotherapies. Protein kinase inhibitors used for combination therapy with CAR T cells include inhibitors of the BTK, the mitogen-activated protein kinase (MAPK) signaling pathway, the PI3K-Akt-mTOR pathway, the JAK/STAT pathway, and other single-targeted kinase inhibitors like inhibitors of p21-activated kinases (PAK) or multi-targeted kinase inhibitors (Table 2).

#### 4.3.1. BTK Inhibitors

The irreversible BTK inhibitor ibrutinib is well established in the clinic for the treatment of CLL and MCL patients. Besides its influence on tumor cells, it has an additional effect on T cells. Ibrutinib can mediate an irreversible inhibition of ITK and enhance T_H_1-mediated responses in preclinical studies [69]. Ibrutinib can additionally induce an increase in T cell numbers as well as a decrease in exhaustion marker expression in T cells, and it can alter the distribution of T_H_ cells and T regulatory (T_reg_) cells [133]. Synergistic administration of anti-CD19 CAR T cells and ibrutinib in mice bearing ALL and CLL led to enhanced engraftment, tumor clearance, and survival [92]. Interestingly, CD19-specific CAR T cells produced from T cells derived from ibrutinib-treated patients showed improved in vitro and in vivo expansion [92]. Anti-CD19 CAR T cells in combination with ibrutinib were able to kill MCL cells more efficiently in vitro and to mediate long-term tumor clearance in a mouse xenograft MCL model [93]. In a mouse model of CRS for B cell malignancies, mice treated with anti-CD19 CAR T cells and ibrutinib showed improved overall survival compared to CAR T cell monotherapy [94]. Interestingly, in this CRS mouse model, serum cytokine levels of markers such as tumor necrosis factor alpha (TNF-α) and interferon-gamma (IFN-γ) were reduced in the presence of ibrutinib [94]. Ibrutinib also reduced the production of inflammatory cytokines by both tumor cells and CAR T cells [94]. In addition to this synergistic effect observed in leukemia models, tumor clearance was also enhanced when anti-CD19 CAR T cells were combined with ibrutinib in a mouse model for Burkitt lymphoma [95]. A clinical trial (NCT02640209) evaluated the safety and efficacy of the combination of anti-CD19 CAR T cells with ibrutinib in r/r chronic lymphocytic leukemia/small lymphocytic lymphoma (CLL/SLL) with partial response or stable disease after ibrutinib monotherapy, showing a high rate of sustained responses with the combination therapy [96]. Interestingly, rapid tumor progression was observed after interrupting the treatment with ibrutinib in CLL patients [134], so that continuing the combination of ibrutinib with CAR T cells seems plausible. Patients treated with anti-CD19 CAR T cells after failure of ibrutinib treatment had high response rates [135]. Another clinical anti-CD19 CAR T cell trial treated CLL patients with ibrutinib more than 2 weeks prior to leukapheresis and proceeded with the treatment for more than 3 months after administration of CAR T cells [97]. Simultaneous administration of ibrutinib and anti-CD19 CAR T cells was well tolerated by CLL patients and led to higher rates of minimal residual disease (MRD)-negative response [97]. In comparison to CAR T cell monotherapy, patients under ibrutinib treatment showed reduced severity of CRS, including reduced CRS-associated cytokine levels together with similar in vivo CAR T cell proliferation [97]. The TRANSCEND CLL 004 clinical trial also combined ibrutinib with the anti-CD19 CAR T cell product lisocabtagene maraleucel for the treatment of r/r CLL/SLL patients [98]. The results showed safety with fewer cases of grade 3 CRS and encouraging responses rates [98]. Despite the low incidence of severe CRS, two fatal cases of cardiac toxicity have been reported, leading to an increased demand for cardiac monitoring in ibrutinib-treated CAR T cell patients with CRS or ICANS [136]. Another clinical trial investigated the influence of concurrent therapy with the BTK inhibitors ibrutinib or acalabrutinib with the anti-CD19 CAR T cell product axi-cel for the treatment of patients with Richter syndrome [99]. Aside from the heterogeneity concerning the administration of BTK inhibitors, the study demonstrated the feasibility and potential of this approach in the therapy of Richter syndrome [99]. The combinatorial approach of CAR T cells with BTK inhibitors will be evaluated in larger clinical trials, and more follow-up data are required for the studies that have already been conducted to better understand the durability of response rates.

#### 4.3.2. Inhibitors of the MAPK Signaling Pathway

The role of the MAPK signaling pathway, especially the activation of Ras, Raf, MAPK kinase (MEK), and extracellular signal-regulated kinase (ERK) proteins, is well described in many malignant diseases. Inhibition of the MAPK pathway (also known as Ras-Raf-MEK-ERK pathway) showed promising results in the treatment of melanoma patients [137]. However, no long-term responses were observed [137]. It is less well known that inhibition of the MAPK pathway also has an enormous impact on T cell function [138]. Indeed, combined administration of TCR-modified T cells and vemurafenib led to enhanced tumor clearance in a BRAF_V600E_-driven murine model of melanoma [100]. A pilot feasibility clinical trial investigated the effect of the combination of TILs with the BRAF inhibitor vemurafenib in patients with metastatic melanoma [101]. Vemurafenib in a high serum concentration negatively influenced the proliferation and viability of both treatment TILs and peripheral blood T cells in vitro; however, clinical results were comparable to TIL therapy without vemurafenib [101]. Approved MAPK pathway-targeted therapies for melanoma such as the BRAF inhibitor vemurafenib, the MAPK inhibitor dabrafenib, and the MEK inhibitor trametinib caused an inhibition of anti-GD2 CAR T cells in vitro at high concentrations [102]. An inhibition at physiological concentrations was only observed with vemurafenib and slightly when dabrafenib was combined with trametinib [102]. Dabrafenib mediated no or weak T cell inhibition at therapeutic-like concentrations and might be a potential candidate for combination therapy with CAR T cells [102]. These observations were confirmed by another group describing no suppression of T cell function by dabrafenib, but partial inhibition of T cell proliferation by trametinib, which can be reversed when combined with dabrafenib [139]. However, trametinib treatment improved tumor-directed immune responses in vivo, highlighting the need for in vivo models to investigate combination strategies with CAR T cells [139]. Another target for the regulation of the Ras-Raf-MEK-ERK pathway is diacylglycerol kinase (DGK). Human anti-FAB or anti-mesothelin CAR T cells intravenously injected into immunodeficient mice bearing subcutaneous mesothelin-positive tumors and then isolated from these tumors showed a loss of functionality limiting their anti-tumor cytotoxicity [140]. Reversibility of this loss of function was seen in the absence of the tumor [140]. An upregulation of intrinsic T cell inhibitory enzymes such as DGK and phosphatase SHP-1 was observed in these hypofunctional T cells [140]. The enzymes DGKα and ζ are highly expressed in T cells and metabolize the second messenger diacylglycerol (DAG), thus acting as regulators of the Ras-Raf-MEK-ERK activation pathway [103]. Indeed, DGK-deficient CAR T cells mediated increased tumor clearance compared to conventional CAR T cells [103]. Additionally, DKG inhibition enhanced CAR T cell functionality [103]. Consequently, inhibition of the Ras-Raf-MEK-ERK pathway has the potential to improve anti-tumor responses mediated by CAR T cells. However, detailed evaluation of this combinatorial approach is still missing.

#### 4.3.3. Inhibitors of the PI3K-Akt-mTOR Signaling Pathway

The PI3K-Akt-mTOR signaling pathway is crucial for several physiological processes and has an influence on both T cells and tumor cells. Rapamycin, an mTOR inhibitor, can ameliorate antigen-specific T cell activity, but also mediates an inhibition of terminally differentiated T effector-like (T_E_) cells. Rapamycin-resistant anti-CD19 CAR T cells maintained mTOR signaling and showed sufficient proliferative capacity and functionality, including cytotoxicity and cytokine production in presence of rapamycin [104]. Remarkably, the combination of rapamycin-resistant anti-CD19 CAR T cells with rapamycin led to enhanced in vitro cytotoxicity against B cell ALL and Burkitt’s lymphoma cell lines compared to rapamycin monotherapy or treatment with conventional anti-CD19 CAR T cells [104]. This approach underlines the potential of mTOR targeting for CAR T cell therapy.

#### 4.3.4. Inhibitors of the JAK/STAT Signaling Pathway

Another important signaling pathway is the JAK/STAT pathway, which plays an essential role for CRS-associated cytokines. The JAK1/2 inhibitor ruxolitinib can prevent CRS after treatment with anti-CD123 CAR T cells without hampering CAR T cell efficacy in a xenograft model by reducing inflammatory cytokines [105]. The selective JAK1 inhibitor itacitinib can reduce the release of cytokines associated with CRS in vitro and in vivo without impairing expansion and antitumor efficacy of anti-GD2, anti-EGFR, and anti-CD19 CAR T cells [106]. Detailed analysis of the influence of inhibitors of the JAK/STAT signaling pathway on CAR T cells still have to be performed.

#### 4.3.5. Inhibitors of the p21-Activated Kinases

A major characteristic of solid tumors is an aberrant vasculature leading to the tumor microenvironment. p21-activated kinase 4 (PAK4) was identified as a selective regulator of genetic reprogramming and aberrant vascularization in glioblastoma-derived endothelial cells [107]. PAK4 deficiency in endothelial cells mediated reconditioning of the tumor microenvironment, normalization of the tumor vasculature, and improvement of T cell migration into tumors [107]. In vivo treatment of mice bearing EGFRvIII-expressing glioblastoma with the PAK4 inhibitor KPT9274 led to sensitization of the tumors to the cellular immunotherapy with enhanced tumor clearance and survival compared to CAR T cells or KPT9274 treatment alone [107]. However, this approach still needs to be studied with CAR T cells targeting other tumor antigens.

#### 4.3.6. Multikinase Inhibitors

Multi-targeted kinase inhibitors are interesting for combination therapy with CAR T cells, as their off-target effects on non-neoplastic cells, especially on T cells, are not fully explored. Sunitinib is a small-molecule, multi-targeted receptor tyrosine kinase inhibitor used for the treatment of renal cell carcinoma (RCC) and imatinib-resistant gastrointestinal stromal tumor (GIST). It can mediate a reduction in immune regulatory cells such as T_reg_ cells and has a positive influence on T cell functionality and migration [108]. Sunitinib mediates an increased surface expression of carbonic anhydrase IX (CAIX) on renal cancer cells [108]. The combination of sunitinib and CAIX-specific CAR T cells administered in mice bearing lung metastases of human RCC showed synergistic efficacy, enhanced proliferative capacity, and migration into the tumor compared to anti-CAIX CAR T cells or sunitinib treatment alone [108]. More pre-clinical data are required to further evaluate this approach.

### 4.4. Apoptosis Regulators

Another strategy for cancer therapy is the combination of drugs utilizing several apoptosis pathways to avoid drug resistance and to induce tumor cell death. Combination of T cells with apoptosis blocking inhibitors, in particular, inhibitors of anti-apoptotic B cell lymphoma 2 (Bcl-2) family proteins, that have a high expression on malignant cells, seems promising (Table 2). B cell tumors are more resistant to therapy due to an upregulation of these Bcl-2 family members, which inhibit intrinsic apoptosis pathways [109]. ABT-737, a Bcl-2 inhibitor, used either as a pre-treatment or synergistically with anti-CD19 CAR T cells derived from healthy donors or from B cell ALL patients, enhanced tumor cell apoptosis [109]. Pre-sensitization of CD19+ tumor cells prior to anti-CD19 CAR T cell therapy with the Bcl-2 inhibitor venetoclax or with the Mcl-1 inhibitor S63845 led to higher target antigen expression and an upregulation of pro-apoptotic proteins in tumor cells, which consequently improved the killing and proliferation of CAR T cells [110]. However, synergistic CAR T cell treatment with these two inhibitors adversely affected CAR T cell numbers and must thus be performed with caution [110]. More investigation of the combination of apoptosis regulators with CAR T cells, especially for solid tumor models, is required.

### 4.5. Epigenetic Modulators

Epigenetic modulators selectively targeting DNA methyltransferases and histone modifying enzymes can control gene expression through transcriptional regulation. This function can also be used for combination therapies with CAR T cells. Interesting epigenetic modulators for combination therapy with CAR T cells include inhibitors of BET bromodomain proteins, of cyclin-dependent kinases (CDK) regulating the cell cycle, and of histone-modifying enzymes (Table 2).

#### 4.5.1. BET Bromodomain Inhibitors

Genomic and transcriptomic analysis of anti-EGFR CAR T cell-treated glioblastoma cells showed an upregulation of genes for inhibitory immune checkpoints, inflammatory cytokines, and immunosuppressive molecules, limiting CAR T cell efficacy [111]. The epigenetic modulator BRD4, a member of the BET subfamily of human bromodomain proteins, was found to be necessary for the activation of these immunosuppressive genes [111]. Therefore, activation of these immunosuppressive genes could be prevented using the BRD4 inhibitor JQ1 [111]. Combination of anti-EGFR CAR T cells with JQ1 led to decreased growth and metastasis of glioblastoma cells and improved overall survival of treated mice compared to CAR T cell or JQ1 treatment alone [111]. Co-treatment with JQ1 mediated a re-sensitization of CAR T cell-resistant tumor cells to CAR T cell therapy [111]. Evaluation of other BET bromodomain inhibitors for combination therapy is missing.

#### 4.5.2. CDK Inhibitors

Transcriptomic analysis of mice bearing a triple-negative breast cancer with an acquired resistance to anti-EGFR CAR T cells showed that treatment with EGFR-specific CAR T cells induced immunosuppressive genes that were associated with CAR T cell-activated enhancers [112]. Screening a panel of epigenetic modulators revealed that these enhancers were sensitive to the selective and potent covalent cyclin-dependent kinase 7 (CDK7) inhibitor THZ1. As THZ1 can suppress anti-EGFR CAR T cell-induced immunosuppressive genes, the combination of THZ1 with CAR T cells was investigated, revealing an enhanced in vitro efficacy compared to anti-EGFR CAR T cells or THZ1 alone [112]. In vivo combination therapy of EGFR-specific CAR T cells with the CDK7 inhibitor THZ1 showed a suppression of immune resistance, tumor growth, and metastasis in triple-negative breast cancer models [112]. Further evaluation of this approach is required.

#### 4.5.3. Histone Deacetylase Inhibitors

Histone acetylation and deacetylation are essential components of gene regulation. Histone regulation is mediated by the complementary effects of histone deacetylases (HDACs) and histone acetyltransferases (HATs); the balance between these two enzyme activities regulates gene expression [113]. Inhibitors of HDACs can induce apoptosis of cancer cells as well as an increase in antigen expression, leading to a significant antitumor activity [141]. Panobinostat, an inhibitor of most HDACs, received FDA approval for the treatment of MM [141]. Panobinostat also mediated reduced growth of pancreatic cancer in a xenograft mouse model [142]. Furthermore, it led to improved proliferation and persistence of adoptively transferred T cells in a melanoma mouse model [143]. These findings underline the rationale to combine panobinostat with CAR T cells. The combination therapy of panobinostat with dual-specific murine CAR T cells expressing a HER2-targeted CAR and a gp100-targeted TCR led to superior tumor eradication of HER2+ pancreatic tumors [113]. Panobinostat also mediated an increased CAR T cell gene accessibility and a more central memory-like T cell phenotype [113]. The same approach with human dual-specific CAR T cells led to similar effects, including enhanced tumor eradication in human pancreatic cancer xenograft mouse models [113]. This concept requires more in vitro and in vivo assays to further understand the underlying mechanisms.

These findings demonstrate that transcriptional modulation using epigenetic modulators is a very promising strategy to overcome immune resistance induced by CAR T cell therapy. However, clinical data are still missing.

### 4.6. Cytokine Inhibitors

Another possible target for combination therapy with CAR T cells is the inhibition of cytokines. The aim could be to influence CAR T cell functionality directly or by modifying the tumor microenvironment. Inhibition of the granulocyte-macrophage colony-stimulating factor (GM-CSF) and of TGF-β seems to be promising for combination therapy with CAR T cells (Table 2).

#### 4.6.1. GM-CSF

Macrophages and monocytes can contribute to the development of CAR T cell-associated CRS and neurotoxicity [114,144,145]. Neutralization of macrophage and monocyte activating cytokines GM-CSF by the humanized monoclonal antibody lenzilumab led to a reduction in myeloid and T cell infiltration in the central nervous system [114]. Co-treatment of anti-CD19 CAR T cells with lenzilumab prevented CRS and reduced neuroinflammation in a special patient acute lymphoblastic leukemia xenograft model without impairing CAR T cell function [114]. Indeed, proliferation of anti-CD19 CAR T cells was improved and response rates in patient-derived xenografts were enhanced after neutralization of GM-CSF by lenzilumab [114]. Moreover, CAR T cells deficient in GM-CSF by CRISPR/Cas9 knockout [146] demonstrated superior in vivo effector function and survival [114]. This approach must be further investigated in clinical trials.

#### 4.6.2. TGF-β

The immunosuppressive character of the tumor microenvironment is upheld—among others—by TGF-β. The combination of CAR T cells targeting the receptor tyrosine kinase-like orphan receptor 1 (ROR1) antigen, which is used for targeted immunotherapy of triple-negative breast cancer, with the TGF-β inhibitor SD-208 led to protection from the immunosuppressive influence of TGF-β and therefore sustained in vitro functionality of CAR T cells [115]. Another side effect was improved CAR T cell viability with a less exhausted phenotype mediated by a reduced PD-1 expression [115]. The concept could also be confirmed with CD133- and HER2-specific CAR T cells using the TGF-β receptor I inhibitor galunisertib [116]. This combination led to superior in vitro killing and cytokine production [116]. Blocking the Wnt pathway using the Wnt inhibitor hsBCL9_CT_-24 can inhibit the expression of TGF-β1 [147]. Combining anti-EpCAM CAR T cells with the Wnt inhibitor hsBCL9_CT_-24 led to enhanced in vitro and in vivo CAR T cell efficacy [117]. This effect can be attributed to a modulation of the tumor microenvironment, a superior tumor infiltration as well as a positive influence on the differentiation and exhaustion status of CAR T cells [117]. These findings might improve CAR T cell therapy in solid tumors. However, detailed in vivo data are missing to further evaluate this approach.

### 4.7. Cyclooxygenase Inhibitors

Besides the inhibition of important protein kinases, other enzymes might also be potential targets for combination therapy with CAR T cells (Table 2). Cyclooxygenase (COX)-inhibitors are widely used as analgesic, anti-pyretic, and anti-inflammatory drugs for many indications. Preclinical and clinical studies demonstrated that the selective COX-2 inhibitor celecoxib might play a promising role in preventing and treating cancer [148]. Therefore, combining CAR T cells with COX-inhibitors seemed quite promising. However, the combination of celecoxib or the non-selective COX inhibitor aspirin together with anti-CD19-CAR T cells should be avoided, as a decrease in quantity and quality of CAR T cells was observed [118]. With both COX inhibitors, CAR T cell activation and proliferation were hampered through a reduction in NF-ĸB signaling [118]. Furthermore, in an antigen stress assay, these COX-inhibitors mediated CAR T cell exhaustion [118]. Therefore, combination of CAR T cells with COX-inhibitors cannot be recommended.

### 4.8. Adenosine Receptor Modulators

Immunosuppressive metabolites in the tumor microenvironment induced by immunomodulatory pathways can be directly targeted to improve CAR T cell efficacy. Targeting the CD73/adenosine pathway is another interesting approach for combination therapy with CAR T cells (Table 2). Adenosine represents a very important immunosuppressive metabolite in the tumor microenvironment of solid tumors [149]. While both adenosine A_2A_ and A_2B_ receptors are upregulated in human CAR T cells, adenosine-mediated suppression of CAR T cell effector function was mediated by the adenosine A_2A_ receptor [149]. Activation of CAR T cells led to enhanced adenosine A_2A_ receptor expression and therefore resulted in CAR T cell suppression [119]. Interestingly, adenosine produced by the tumor cells activates adenosine A_2A_ receptors and therefore inhibits anti-tumor-directed T cell responses [119]. The disruption of the adenosine A_2A_ receptor gene by CRISPR/Cas9 prevented exhaustion and improved effector function of anti-mesothelin CAR T cells [149]. These adenosine A_2A_ receptor knock-out mesothelin-specific CAR T cells demonstrated superior in vivo antitumor efficacy [149]. Additionally, pharmacological or genetic targeting of adenosine A_2A_ receptors improved the efficacy of anti-HER2 CAR T cells by increasing activation and cytokine production, especially when additionally combined with PD-1 blockade [119].

Furthermore, modulation of the adenosine A_2B_ receptor can be used for combination therapy with CAR T cells. The adenosine A_2B_ receptor agonist BAY 60-6583 combined with anti-CD133 or anti-HER2 CAR T cells led to an enhanced proliferative capacity, cytotoxicity, and cytokine production in in vitro assays [120]. This combination also improved tumor clearance by anti-HER2 CAR T cells in a xenograft mouse model [120]. Detailed analysis of the effects revealed that BAY 60-6583 improved T cell function through mechanisms independent of the adenosine A_2B_ receptor [120].

Targeting the adenosine A_2A_ and A_2B_ receptors together with CAR T cells has a high translational potential for cancer therapy. Further investigation of the role of adenosine receptor agonists and antagonists for the combination with CAR T cells is mandatory.

## 5. Discussion

Preclinical and clinical data suggest that combination of several monoclonal antibodies or small molecule inhibitors with CAR T cells may enhance clinical response rates in a broad range of cancers. Repurposing already approved drugs for a new indication represents an encouraging new field of research. A major advantage is cost reduction of drug development compared to de novo drug development and the much faster path to receive approval for already approved drugs in other settings. For the choice of the compound, it would be optimal to attack the tumor, e.g., by targeting a driver mutation while at the same time positively influencing CAR T cells. This dual therapeutic effect on both tumor and T cells has great potential to overcome current limitations of CAR T cell therapy.

Several approaches of expanding CAR T cells ex vivo in the presence of small molecule inhibitors have been presented. The most promising combination partners are protein kinase inhibitors, notably PI3K inhibitors, due to their ability to modify the composition of T cell subsets and to induce a less differentiated and less exhausted T cell immunophenotype. In the early years of CAR T cell therapy, it was assumed that the therapeutic success depends on the amount of transfused CAR T cells. However, after reaching a certain threshold, in vivo proliferation and response rate do not directly correlate with transfused cell numbers [29]. Immunophenotype and cellular composition of the final CAR T cell product have a major influence on the therapeutic success of this T cell-based immunotherapeutic approach [150,151]. Even if cytotoxic CD8+ T cells play a leading role in the killing of tumor cells, CD4+ T_H_ cells are also very important and highly potent T cells [152]. A balanced ratio of CD8+ T cells to CD4+ T cells is beneficial for anti-tumor efficacy [13,15]. To achieve a balanced ratio, subsets must be isolated and produced separately, leading to a more complicated CAR T cell production process. PI3K inhibitors can positively influence the CD8+ to CD4+ T cell ratio [51]. The T cell differentiation also influences the success of CAR T cell therapy. Terminally differentiated T_E_ cells mediate superior in vitro anti-tumor efficacy; however, activation, expansion, and persistence were impaired in vivo [14]. These findings shifted the focus of T cell subpopulations towards less-differentiated T cells for adoptive T cell therapy. Less-differentiated T_N_ cells and T_SCM_ cells can expand and persist for a long time after administration into patients and have the potential to mediate a long-lasting response [16]. Ex vivo expansion of CAR T cells with protein kinase inhibitors can mediate this favorable T cell phenotype. Patients with a high exhaustion marker expression are more likely to not respond or to have an early relapse after adoptive T cell therapy [66]. CAR T cells expanded with PI3K inhibitors have the potential to overcome these limitations linked to excessive exhaustion status. Complicated manufacturing processes and production failures are also important limitations of adoptive T cell therapy. Addition of PI3K inhibitors during ex vivo T cell expansion can potentially reduce manufacturing time and failure rates. However, the manufacturing process of CAR T cells might also become more complicated and more expensive compared to conventional production if an additional compound is integrated into the process.

In particular, the strategy of concurrent combination of CAR T cells with immune checkpoint modulators seems to be a very promising and feasible approach. The strategy is already being tested in clinical phase I trials. A major advantage of this approach is that immune checkpoint modulators are already FDA approved. However, the treatment of patients with antibodies commonly requires multiple administrations [123]. Further, the fact that antibodies possess variable pharmacokinetic characteristics and abilities to infiltrate solid tumors could limit this promising approach [123]. Additionally, systemic non-targeted application could lead to toxicity, limiting the feasibility and safety [123]. Cell-intrinsic strategies for immune checkpoint blockade might overcome these limitations. Transduction of human T cells with a bicistronic lentiviral vector for anti-CAIX CAR and anti-PD-L1 scFv antibodies led to less exhaustion and improved functionality of CAR T cells in an orthotopic RCC model [153]. Enhanced in vivo function was also achieved with anti-PD-1-blocking scFv secreting anti-CD19 CAR T cells [154,155]. Another intrinsic strategy is to equip CAR T cells with a dominant negative receptor for PD-1, leading to an increased effector function [124]. Genome editing can be used to confer resistance to PD-1 signaling [123]. With cell-intrinsic strategies, multiple administrations of antibodies can be reduced to a single administration. Nonetheless, it is not yet clear if a cell-extrinsic or a cell-intrinsic approach is less toxic and more effective [123]. Additionally, other inhibitory receptors can be upregulated on T cells after chronic stimulation [156], thus potentially making PD-1 blockade insufficient [122,157] and highlighting the need for other blocking antibodies as combination partners [123]. Multiple trials are currently investigating the combination of CAR T cells with immune checkpoint modulators to further understand the impact of this promising approach for the future of CAR T cell therapy.

## 6. Conclusions

The adoptive transfer of CAR T cells has revolutionized modern care of patients suffering from distinct hematological entities. Patients with CD19-positive and BCMA-positive malignancies profit from this cellular therapy. For CAR T cells that target other tumor antigens, especially tumor antigens expressed in solid tumors, however, improvements are still necessary. Therefore, a major focus for optimization of CAR T cell therapy is enabling broad CAR T cell therapy in solid tumors as well as tumor antigen specificity to reduce therapy-associated toxicity. An increase in CAR T cell efficacy might be achieved by administration of an improved final CAR T cell product with a favorable phenotype. This might be possible by ex vivo expansion of CAR T cells in the presence of small molecule inhibitors. Another approach is the concurrent in vivo combination therapy of CAR T cells with other drugs. Combination therapy with CAR T cells is an evolving research field with many clinical trials ongoing. However, obstacles such as costs and feasibility need to be further addressed. Optimal conditions for CAR T cell production and administration are yet to be defined. Therefore, further efforts are obligatory for optimizing CAR T cell production protocols and treatment regimes.

## Figures and Tables

**Table 1:** Summary of compounds used for ex vivo treatment of T cells.

Drug Class	Compound	T Cell Product	Ref.
Protein kinase inhibitors			
mTOR inhibitor	Rapamycin	T cells	[46]
		Anti-EpCAM CAR T cells	[47]
Akt inhibitor	Akt inhibitor VIII	TILs	[48]
			[49]
		Anti-CD19 CAR T cells	[50]
PI3K inhibitor			[51]
	Idelalisib		[52]
			[53]
	Eganelisib	Anti-mesothelin CAR T cells	
	Umbralisib		
	Duvelisib		
	LY294002	Anti-CD33 CAR T cells	[54]
	Idelalisib	CAR T cells	[55]
	Duvelisib		
	bb007	Anti-BCMA CAR T cells	[56]
	Duvelisib	Anti-CD5 CAR T cells	[57]
	Idelalisib		
BTK inhibitor	Ibrutinib	Anti-CD19 CAR T cells	[58]
**Hormone receptor inhibitors**			
VIP receptor antagonist	VIPhyb	Anti-CD5 CAR T cells	[57]
**Epigenetic modulators**			
	JQ-1	Anti-CD19 CAR T cells	[59]
BET bromodomain inhibitor		Anti-CD33 CAR T cells	[60]
	iBET		
**Immunomodulatory drugs**			
Immunomodulator	Lenalidomide	Anti-CS1 CAR T cells	[61]

**Table 2:** Summary of synergistic combination partners.

Drug Class	Compound	T Cell Product	Ref.
Immune checkpoint modulators			
Anti-PD-1 antibody	Clone RMP1-14	Anti-HER2 CAR T cells	[73]
	Pembrolizumab	Anti-GD2 CAR T cells	[74]
		Anti-CD19 CAR T cells	[75,76,77]
		Anti-GD2 CAR T cells	[78]
		Anti-mesothelin CAR T cells	[79]
	Nivolumab		[80,81]
Anti-PD-L1 antibody	Atezolizumab	Anti-CD19 CAR T cells	[82]
	Durvalumab		[83]
Anti-CTLA-4 antibody	Ipilimumab		
Anti-4-1BB antibody	Clone 3H3	Anti-HER2 CAR T cells	[84]
	Utomilumab	Anti-CD19 CAR T cells	[85]
**Immunomodulatory drugs**			
Immunomodulator	Lenalidomide	Anti-BCMA CAR T cells	[86]
		Anti-CS1 CAR T cells	[61]
		Anti-CD20 CAR T cells	[87]
		Anti-CD19 CAR T cells	[87,88]
		Anti-CD133 CAR T cells	[89]
		Anti-HER2 CAR T cells	
		Anti-EGFRvIII CAR T cells	[90]
		Anti-WT1 CAR T cells	[91]
**Protein kinase inhibitors**			
BTK inhibitor	Ibrutinib	Anti-CD19 CAR T cells	[92,93,94,95,96,97,98,99]
	Acalabrutinib		[99]
BRAF inhibitor	Vemurafenib	OT-1 TCR-engineered T cells	[100]
		TILs	[101]
	
MAPK inhibitor	Dabrafenib	Anti-GD2 CAR T cells	[102]
MEK inhibitor	Trametinib		
DGK inhibitor	DGK1 + DGK2	Anti-mesothelin CAR T cells	[103]
mTOR inhibitor	Rapamycin	Anti-CD19 CAR T cells	[104]
JAK inhibitor	Ruxolitinib	Anti-CD123 CAR T cells	[105]
	Itacitinib	Anti-CD19 CAR T cells	[106]
		Anti-GD2 CAR T cells	
		Anti-EGFR CAR T cells	
PAK inhibitor	KPT9274	Anti-EGFRvIII CAR T cells	[107]
Multikinase inhibitor	Sunitinib	Anti-CAIX CAR T cells	[108]
**Apoptosis regulators**			
Bcl-2 inhibitor	ABT-737	Anti-CD19 CAR T cells	[109]
	Venetoclax		[110]
Mcl-1 inhibitor	S63845		
**Epigenetic modulators**			
BET bromodomain inhibitor	JQ-1	Anti-EGFR CAR T cells	[111]
CDK inhibitor	THZ1		[112]
Histone deacetylase inhibitor	Panobinostat	Dual-specific CAR T cells	[113]
**Cytokine inhibitors**			
GM-CSF inhibitor	Lenzilumab	Anti-CD19 CAR T cells	[114]
TGF-beta inhibitor	SD-208	Anti-ROR1 CAR T cells	[115]
	Galunisertib	Anti-CD133 CAR T cells	[116]
		Anti-HER2 CAR T cells	
**Inhibitors of Wnt signaling**			
Wnt inhibitor	hsBCL9_CT_-24	Anti-EpCAM CAR T cells	[117]
**Cyclooxygenase inhibitors**			
Non-selective COX inhibitor	Aspirin	Anti-CD19 CAR T cells	[118]
Selective COX-2 inhibitor	Celecoxib		
**Adenosine receptor modulators**			
Selective adenosine A_2A_ receptor antagonist	SCH58261		[119]
	ZM241385	Anti-HER2 CAR T cells	
Selective adenosine A_2B_ receptor agonist	BAY 60-6583		[120]
		Anti-CD133 CAR T cells	
